# IPF patients are limited by mechanical and not pulmonary-vascular factors – results of a derivation-validation cohort study

**DOI:** 10.1186/s12890-019-1015-3

**Published:** 2019-12-11

**Authors:** Benjamin D. Fox, Yael Shostak, Barak Pertzov, Baruch Vainshelboim, Shimon Itzhakian, Irit Terner, Mordechai R. Kramer

**Affiliations:** 1 Pulmonary Institute, Rabin Medical Center, Petach Tikva, Israel; 2 Pulmonary Institute, Shamir Medical Center, Tzrifin, Israel; 3 Sackler Faculty of Medicine, Tel Aviv University, Tel Aviv, Israel

**Keywords:** Idiopathic pulmonary fibrosis, Exercise, Cardiopulmonary exercise test, Pulmonary hypertension

## Abstract

**Background:**

During cardiopulmonary exercise testing (CPET), Idiopathic Pulmonary Fibrosis (IPF) patients do not reach their direct maximum voluntary ventilation (MVV) and have deranged gas exchange. Their exercise limitation is therefore attributed to a pulmonary vascular mechanism.

**Methods:**

We studied two cohorts (derivation and validation) of IPF patients with lung function testing and CPET. Maximal ventilation at exercise (VEpeak) was compared to direct MVV by Bland-Altman analysis.

**Results:**

In the derivation cohort (*n* = 101), direct MVV over-estimated VEpeak by a factor of 1.51, driven by respiratory rate during MVV that was 1.99 times higher at rest as compared to VEpeak at exercise. The formula (FEV1 × 20.1) + 15.4 was shown to predict VEpeak (r^2^ = 0.56) in the derivation cohort. In the validation cohort of 78 patients, VEpeak was within a factor of 1.27 (6.8 l/min) of predicted according to the novel formula. According to the novel prediction formula the majority of patients (58%) in the entire cohort have VEpeak within 85% of their predicted MVV, which would indicate a mechanical respiratory limitation to exercise.

**Conclusion:**

Estimation of direct MVV performed at rest leads to significant over-estimation of the breathing reserve in IPF patients. This may lead to over-diagnosis of pulmonary vascular limitation in these patients. Expected maximal ventilation at exercise may be accurately predicted indirectly by an IPF-specific formula.

## Background

Symptoms of exercise-related dyspnea are almost ubiquitous in patients suffering from idiopathic pulmonary fibrosis (IPF) [1]. In resting lung function testing, restrictive deficits in lung volumes and impaired diffusion capacity are characteristic findings. During field exercise tests like the 6 min walk distance and step climbing, decreased exercise capacity and hypoxemia are evident [2]. It is possible to further quantitate functional capacity and causes of exercise limitation by means of the cardiopulmonary exercise test (CPET) [3, 4]. To assess respiratory function during exercise, the key parameter is Ventilation at peak exertion (VEpeak) compared to a value estimated from resting lung function testing. The expected value for a given individual may be measured *directly* by asking the patient to perform a maximal hyperventilation over 12 seconds, which is multiplied by 5 to determine the maximum voluntary ventilation (direct MVV) over 1 minute [3, 4]. Maximal ventilation at exercise may also be estimated *indirectly* - in patients with normal lungs or chronic obstructive pulmonary disease, FEV1 (forced expired volume in 1 second) multiplied by 35 approximates the MVV quite well (the indirect MVV method) [5–9]. Subjects whose VEpeak is above 85% of their direct or indirect MVV are determined to have a *respiratory mechanical limitation* to exercise [3].

It has been noted that during the short MVV maneuver, IPF patients may be able to generate higher levels of respiratory rate and ventilation than they are able to do during the subsequent bout of exercise. Remarkably this has not been evaluated beyond anecdotal comments [3]. Furthermore, the indirect FEV1 × 35 formula has not been validated in IPF patients. The implication of this is that IPF patients, since they do not reach the predicted respiratory mechanical limit but have derangements in gas exchange, according to the diagnostic logic of CPET interpretation, they have a *pulmonary vascular limitation* [3, 10]. Subsequent studies have generally confirmed that exercise capacity is reduced in IPF patients who have evidence of pulmonary hypertension (PH) compared to those without, further supporting this hypothesis, however not all studies are consistent [11–16]. The most in-depth physiological study by Degani-Costa et al. studied IPF patients with invasive hemodynamic monitoring during exercise [14]. These authors demonstrated that patients with IPF and PH have worse exercise capacity and gas exchange derangement than IPF patients without PH. However, given that many patients did not have PH at rest or at exercise yet still had significant exercise limitation, it strongly suggests that other factors may play a role.

We hypothesized that IPF patients do have a respiratory mechanical limitation, yet the direct MVV maneuver at rest sets the threshold for diagnosing that limitation at an unobtainable high level for IPF patients, therefore making the respiratory mechanical limit almost impossible to reach and directing the interpreting physician to diagnose pulmonary vascular limitation. The aim of the present study was to (a) evaluate whether direct MVV systematically overestimates the VEpeak reached by IPF patients and (b) to derive and validate an IPF-specific indirect MVV formula for CPET based on resting lung function measurements.

## Methods

The study was approved by the hospital research ethics committee. All subjects were diagnosed with IPF according to accepted clinical-radiological-pathological diagnostic criteria [1]. All data were collected during routine clinical practice. Typically CPET examinations took place as part of pre-lung transplantation workup or at the request of their treating pulmonologist. Exclusion criteria were severe cardiovascular or musculoskeletal limitation as judged by the investigators. All patients performed spirometry, direct MVV, body plethysmography and single breath diffusion capacity for carbon monoxide (DLCO) in accordance with ATS/ERS guidelines (ZAN-600 system, Inspire) [17, 18]. Cardiopulmonary exercise testing was performed on a ZAN-500 cycle ergometery CPET system with a 10-15 W/min increment, as determined by the clinical judgment of the referring physician. Patients exercised until they reached their maximum exertion based on their perception or reached maximal heart rate, respiratory reserve or developed one of the accepted safety events for stopping an exercise test [4]. Two cohorts of patients were studied - derivation and validation, as described below in the statistical plan.

### Statistical plan

Summary statistics were reported as mean (standard deviation) or counts as appropriate. The primary endpoint of the study was the bias and limits of agreement between VEpeak measured during CPET with both direct MVV or indirect MVV using the Bland-Altman plot [19]. In cases of proportional bias we log-transformed the data before analysis. From the first (derivation) cohort we derived a regression formula for an indirect MVV estimation based on standard lung function tests and VEpeak by linear regression. Subsequently we recruited a second (validation) cohort. In this cohort we repeated the initial Bland-Altman analysis for direct MVV versus actual VEpeak and also performed a similar analysis with the novel indirect MVV prediction formula versus actual VEpeak achieved. In an exploratory analysis we categorised each subject as reaching a respiratory mechanical limitation if VEpeak reached 85% of predicted by either direct MVV or according to the novel indirect MVV regression formula [3]. In a second exploratory analysis, analyzed the ratio of inspiratory capacity at rest to tidal volume at peak exertion, to further assess whether patients were mechanically limited or not.

Sample size was estimated at 80–100 patients per cohort, as described by Collins et al., in their paper on validation of clinical prediction rules [20].

## Results

We recruited 98 patients into the derivation cohort and 73 patients into the derivation cohort. Demographic and physiological characteristics of the patients at rest and at exercise are shown in Table 1.

**Table 1:** Patient characteristics and results of lung function and cardiopulmonary exercise tests

	Derivation		Validation	
	Absolute	% Predicted	Absolute	% Predicted
N	98	–	73	–
Age (y)	62(8)	–	64(10)	–
Sex (% Male)	62	–	68	–
BMI (kg/m)	28.6 (4.1)	–	29.7 (15.3)	–
FVC (L)	1.83(0.62)	54(17)	2.14(0.8)*	65(20)*
FEV1 (L)	1.55(0.50) 2	57(18)	1.82(0.66)*	70(22)*
FEV1/FVC ratio	87 (9)		86(7)	
TLC (L)	3.58(0.92)	57(13)	3.74(1.19)	64 (15)*
DLCO (ml/min/mmHg)	10.21(4.05)	38(13)	12.24 (4.78)	51(17)*
Indirect MVV FEV1 × 35 (L/min)	54(18)	–	63(23)	–
Direct MVV (L/min)	67.4(23.0)	–	76(28)*	–
Direct MVV (breaths/min)	94.4(22.7)	–	90(26)	–
Tidal Volume (L)	0.60 (0.25)	–	0.54 (0.15)	–
SpO2 (%)	94 (3)	–	95 (2)	–
Peak Work Rate (W)	57 (34)		72 (32)*	
VO2 (ml/kg/min)	11.5(4.3)	64(14)	11.4 (3.6)	62(19)
RER	1.12(0.13)	–	1.02(0.16)	
VE peak (L/min)	44(14)	–	42(15)	–
Resp Rate (L/min)	47(12)	–	40(10)*	–
Heart Rate (bpm)	149(24)	–	120(21)*	–
Tidal Volume (L)	0.99 (0.38)	–	1.12 (0.45)	–
VE/VCO2 ratio (nadir value)	45 (17)	–	33(6)*	–
O2 Pulse (ml/100 ml blood)	8.1 (3.6)	–	9.7 (3.1)*	
SpO2 (%)	84 (7)	–	87 (6)	–

All continuous data shown as mean (sd). * significant difference between groups (t-test) *p* < 0.05

### Derivation cohort

In the derivation cohort, 98 subjects were studied (62% males, mean age 62 years). Patients were characterized by the typical resting deficits in lung function (FVC 54%, DLCO 48%) and reduced peak oxygen uptake (VO2–11.4 ml/kg/min 64%), Table 1.

**Fig. 1:**
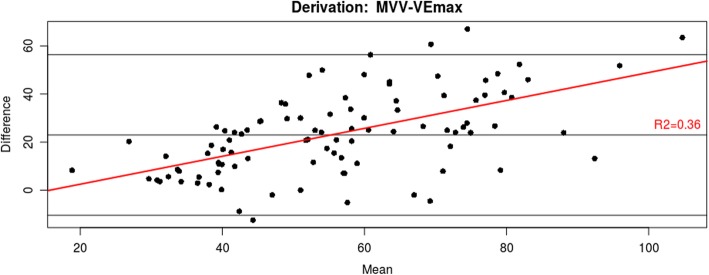
Bland-Altman plot showing predicted VEpeak (direct MVV) and actual VEpeak during CPET examination in the derivation population. Proportional bias is demonstrated (red line)

**Fig. 2:**
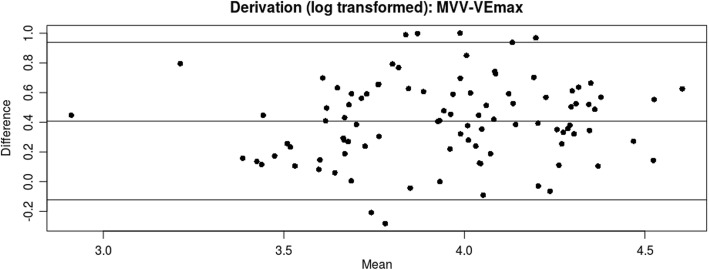
Bland-Altman plot showing predicted VEpeak (direct MVV) and actual VEpeak during CPET examination in the derivation population, after log transformation. Proportional bias is eliminated

For the primary endpoint analysis, direct MVV was seen to overestimate actual VEpeak by + 23.4 L/min (95% CI -10.8 - 57.6). There was evidence of significant proportional bias in the data (Fig. 1), r^2^ = 0.33, *p* < 0.001. After log transformation which removed the proportional bias, MVV was shown to overestimate VEpeak by a factor of 1.51 (95% CI 0.89–2.58), Fig. 2. Indirect MVV (FEV1 × 35) overestimated VEpeak by a factor of 1.21 (95% CI 0.94–2.10). Of note, respiratory rate during the direct MVV procedure was approximately double the respiratory rate achieved at peak exercise - factor of 1.99(95% CI 1.00–3.94).

**Fig. 3:**
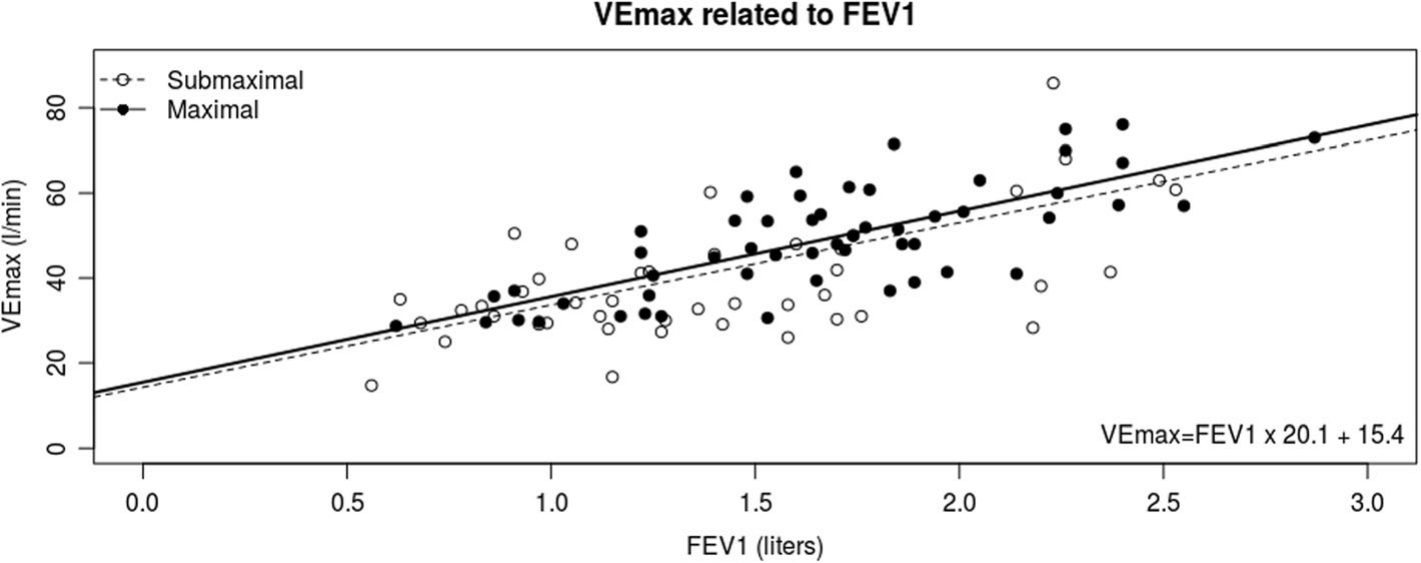
Linear regression showing VEpeak as a function of FEV1

By linear regression, we performed simple univariate linear regression for FEV1, FVC, TLC or MVV as the independent predictor for VEpeak, Table 2. The same analysis was performed with the entire derivation cohort and a subset of 55 patients with peak RER > 1.10 (RER = VCO2÷VO2) who represented the patients who did the most maximal CPET examination and would therefore be expected to have approached more closely their true peak ventilation. The best-fit formula for indirect MVV was (FEV1 × 20.1) + 15.4, (r^2^ = 0.56) derived from the maximal-exertion patients, using the absolute value of FEV1 in Liters, Fig. 3.

**Table 2:** Regression formulas for predicting VEpeak from resting lung function tests in the derivation cohort, sorted in descending order of value of r^2^. All regression formulas used the absolute value not percent of predicted

Covariate	Gradient	Intercept	Adjusted r^2^
Only RER > 1.10			
FEV1	20.1	15.4	0.56
FVC	15.9	16.92	0.55
TLC	8.9	17.8	0.40
MVV	0.35	22.1	0.39
DLCO	0.9	42.1	0.10
Entire Cohort			
FEV1	19.1	14.7	0.48
FVC	15.9	15.3	0.47
MVV	0.42	16.3	0.45
TLC	9.7	12.4	0.33
DLCO	1.1	35.7	0.07

### Effect of pulmonary hypertension on exercise

In the derivation cohort 71 patients had data from right heart catheterization. The other 27 patients had an estimated systolic pulmonary artery pressure from tricuspid jet velocity - for these patients we estimated mPAP = 0.61× sPAP + 2 mmHg [21]. We classified each subject as having PH where mPAP≥25 mmHg. According to this technique 51 of the 98 patients had PH. There was a very weak, although statistically significant negative correlation between VO2 and mPAP (r^2^ = 0.07, *p* < 0.01). In patients with PH, oxygen uptake was significantly reduced compared to patients without PH (10.3 ml/kg/min vs. 12.7 ml/kg/min, respectively, *p* = 0.005).

### Validation cohort

**Fig. 4:**
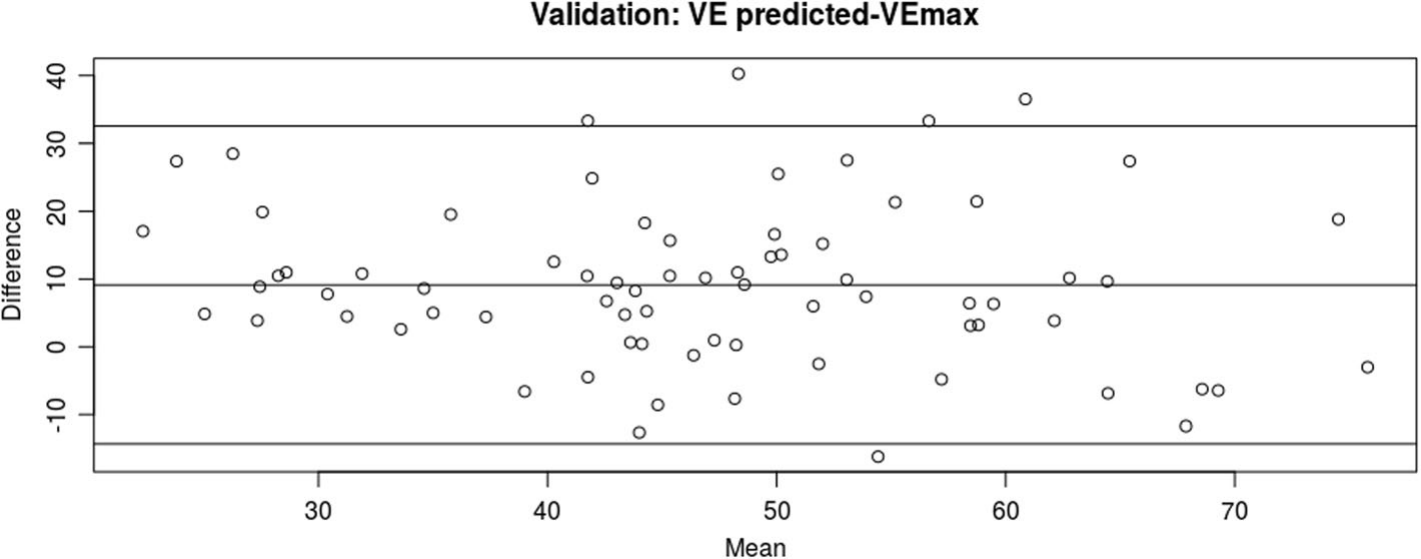
Bland-Altman plot showing predicted VEpeak (VEpeak = FVC × 20.1 + 15.4) and actual VEpeak in the validation cohort

We subsequently recruited a further 73 patients to validate the regression formula described above. Subjects’ age and sex were broadly similar to the derivation cohort, Table 1. However the derivation cohort did appear to be slightly healthier in terms of resting lung function. We repeated the initial analysis and re-demonstrated that direct MVV overestimated VEpeak by a factor of 1.79 (95%CI 0.98–3.3). When compared to the novel indirect MVV prediction formula (FEV1 × 20.1) + 15.4, the Bland-Altman analysis demonstrated an absolute bias of + 9.5 L/min (95%CI -13.8 - 32.9 L/min) above the actual VEpeak achieved during exercise without evidence of proportional bias, Fig. 4. After log-transformation for the purposes of comparison, the derived regression formula overestimated actual VEpeak by a factor of 1.27 (95%CI 0.69–2.33). After selecting only patients with RER > 1.10 the mean bias was reduced: + 6.8 L (95%CI -15.7 - 29.2) or by a factor of 1.17 (95%CI 0.75–1.81).

### Categorization of CPET results with different techniques

In an exploratory analysis, we calculated breathing reserves (MVV-VEpeak) as absolute values in L/min and also as a proportion of MVV with three different techniques – direct MVV, indirect MVV by the traditional formula and indirect MVV with the novel formula (Table 3) [3]. In the validation cohort, the breathing reserve was lowest when the novel indirect MVV formula was used, and the proportion of subjects reaching the mechanical respiratory limit (VEpeak≥85% of predicted) was highest compared to other techniques. We also dichotomized the entire cohort of 171 patients as reaching the respiratory mechanical limit or not at peak exercise, determined by VEpeak 85% of predicted (Table 3). When breathing reserve was predicted from direct MVV, 27/171 (16%) of the subjects would be defined as showing a mechanical limitation. When predicted VEpeak was defined by the novel indirect MVV prediction formula (FEV1 × 20.1) + 15.4, 101/171 (58%) of the subjects reached the mechanical limit - chi square *p* < 0.001. In the subgroup of patients achieving RER > 1.10, the proportion of patients with mechanical limitation by the novel technique was 57/74 (77%).

**Table 3:** Breathing Reserves of the cohorts as defined by direct MVV, traditional indirect MVV formula (FEV1 × 35) and the novel indirect MVV formula ((FEV1 × 20.1) + 15.4)

	Derivation Cohort	Validation Cohort
N measurements	98	73
Measured VEpeak (L/min)	44.3 (14)	42.4 (15)
MVV Technique (L/min)		
1. Direct	67.4 (23.0)	76(28)
BR (L/min)	23.0 (17)	34.2 (21)
VEpeak: MVV	0.68 (0.19)	0.58 (0.18)
Proportion VEpeak≥0.85 MVV	20 [26%]	7 [10%]
2. Indirect: FEV1 × 35	54(18)	63(23)
BR (L/min)	9.8 (13)	21 (17)
VEpeak:MVV	0.86 (0.23)	0.70 (0.21)
Proportion VEpeak≥0.85 MVV	46 [47%]	16 [22%]
3. Indirect: (FEV1 × 20.1) + 15.4	46.5 (10)	51.0 (13.2)
BR (L)	2.1 (10)	9.5 (11.9)
VEpeak:MVV	0.96	0.82 (0.22)
Proportion VEpeak≥0.85 MVV	68 [69%]	33 [45%]
Inspiratory Capacity Analysis		
N measurements	52	44
Inspiratory Capacity (L)	1.39 (0.52)	1.51 (0.62)
VTmax (L)	0.99 (0.38)	1.12 (0.45)
VTmax:IC	0.81 (0.2)	0.76 (0.18)
Proportion VTmax:IC ≥ 0.70	38 [73%]	26 [59%]

### Exploratory analysis: tidal volume:inspiratory capacity ratio as an index of mechanical respiratory limitation

In an exploratory post-hoc analysis, we analyzed the ratio of tidal volume at peak exercise to inspiratory capacity measured at baseline (VTmax:IC ratio). Reports of normal subjects suggest that the typical ratio is about 0.7, whereas in patients with emphysema who experience air-trapping, the ratio is higher [22]. Although the data-set was incomplete due to missing data, in both cohorts, the mean ratio was 0.78. Taking 0.7 as an arbitrary cut-off value, 67% of subjects reached a high VTmax: IC ratio, Table 3.

## Discussion

We performed a systematic derivation-validation cohort study to challenge the paradigm that patients with IPF are limited in exercise by a pulmonary-vascular rather than a respiratory-mechanical limitation. We showed that the traditional threshold values for setting the mechanical respiratory limit derived from resting measurements (direct MVV or FEV1 × 35) significantly over-estimate the level of ventilation that IPF patients can reach during peak exertion. This seems to have been driven by very high respiratory rates during the direct MVV maneuver, which were twice as high as the respiratory rate achieved at maximal exertion. We then derived an IPF-specific prediction formula for indirect MVV, which we validated against a second independent cohort. In the validation cohort our formula predicted MVV to within 6.8 to 9.5 l/min. There was no evidence of proportional bias in the novel indirect MVV formula, suggesting that the formula is valid across the measured range of FEV1 values (Fig. 4). Patients doing more intense exertion who reached higher RER showed a closer agreement to the prediction formula to those who may have ceased exertion below their true physiological maximum.

This work is important as we systematically re-evaluated the seminal paper by Hansen et al. that determined that patients with interstitial lung diseases are not mechanically limited [10]. Whilst it is clear that pulmonary hypertension (PH) exists in some patients with IPF, it is not a ubiquitous finding. PH tends to develop as patients progress towards end-stage disease, such as in cohorts of lung transplantation candidates and it is an adverse prognostic factor [12]. However almost all IPF patients complain of dyspnea on exertion yet not all have PH, so logically PH cannot be the explanation for dyspnea in all IPF patients [12, 14]. Indeed, in the exceptional study by Degani-Costa et al., it was shown that IPF with PH patients did have lower VO2 than the IPF without PH cohort, but in both cohorts, VO2 was significantly decreased and maximal ventilation was at 88% of predicted with no difference between the two groups [14]. This fact also supports the assertion that IPF patients are mechanically limited by their fibrotic non-compliant lungs but those who have developed PH have an additional pulmonary vascular/right ventricular impairment which reduces their exercise capacity even more. We also note that the original work by Hansen was performed in a small case series of 42 mixed interstitial lung disease patients of whom only 9 had idiopathic pulmonary fibrosis as defined at the time [10]. The present study included over 170 IPF patients across both cohorts diagnosed by up-to-date diagnostic criteria, making it one of the largest reported cohorts of CPET in IPF [1]. The logical consequences of an IPF-specific prediction formula are clear when patients were categorized by the traditional or novel criteria. In the traditional direct-MVV-based paradigm, only 16% would be diagnosed with a mechanical limitation, whereas with the novel technique 58% had a mechanical limitation. This proportion rose to 77% when only patients achieving high intensity exercise were examined. These findings strongly support the hypothesis that IPF are actually limited by a mechanical limitation. In an exploratory analysis, we calculated the ratio of VTmax:IC, which has been suggested as a useful index of mechanical limitation in emphysema disease patients. The subjects in our cohort did show a high ratio (mean 0.78) compared to a suggested normal ratio (0.7) in 67% of cases. Again this supports our hypothesis of mechanical limitation in IPF patients but these results should be interpreted very cautiously since (1) there were only data in about half of the subjects, and (2) the limits of normal are not well described for this index and (3) this was a post-hoc exploratory analysis. This should be the subject of future research.

One limitation of the present study is that we did not perform invasive pulmonary hemodynamic measurements during exercise so we cannot completely exclude a pulmonary vascular limitation. Indeed all studies of this topic with the exception of Degani-Costa et al. have used resting hemodynamics which were obtained in a supine posture at a different time to the exercise test. Many patients in the present cohort had PH diagnosed by right heart catheterization or by echocardiography, and we reproduced the deleterious effect of resting pulmonary artery pressure on peak oxygen uptake [11–15]. The ventilatory-equivalent for CO2 (VE/VCO2) in our subjects was increased (Table 1), and patients experienced oxygen desaturation and therefore reduction in O2 pulse. These parameters reflect VQ mismatching in the lung, and the presence of significant VQ mismatch during exercise is typically seen in pulmonary vascular disease [23]. However VQ mismatch also exists in the fibrotic lung due to both parenchymal and vascular disease and therefore the finding of a high VE/VCO2 and desaturation is not specific only for pulmonary vascular disease. In the Degani study, even patients without PH measured by exercise catheterization had increased VE/VCO2 [14]. We suggest that pulmonary hypertension, when present in IPF patients, is an exacerbating factor which further decreases exercise capacity, but that the primary limitation to exercise is mechanical. A second potential limitation is that our cohort contained many severe IPF cases some of whom were in pre-transplantation workup. It could be argued that our sample was biased towards demonstrating mechanical limitation in patients with severe parenchymal disease and reduced FVC, and they may not be representative of milder patients. However, as stated above, it is well documented that PH is uncommon in mild cases of IPF and so we suggest that in milder cases a pulmonary vascular limitation is even *less* likely to exist compared to our cohort. Furthermore, as discussed above, the absence of proportional bias in the validation cohort (Fig. 4) suggest that the formula is valid at low and high values of FEV1.

Our study is also consistent with previous studies. In a small series of interstitial lung disease patients published previously, patients had mean FVC 2.83 L and mean VEpeak 63.8 L/min, where our formula predicts 62 L/min [24]. Similarly, in a recent series, our formula almost exactly predicts the mean VEpeak achieved based on mean FEV1 [25]. Other authors published an indirect MVV prediction for ILD patients VEpeak (FVC × 18.9) + 12, which is strikingly similar to our formula (Table 2) [26]. These three studies give a certain external validity to our data, and we encourage other researchers to evaluate and refine our formula on their own cohorts, including those with milder disease. A final limitation is that we did not formally evaluate potential dynamic hyperinflation (via inspiratory capacity measurements during exercise) or muscle fatigue in our patients. However, a previous study has shown that dynamic hyperinflation is not a typical feature of IPF, and muscle fatigue seems to play only a small role in exercise limitation [25].

## Conclusion

In conclusion, this study suggests that the apparent lack of a respiratory mechanical limitation in IPF patients may be related to an over-estimation of the patients respiratory reserve via the traditional direct or indirect MVV measurements. Maximal ventilation in an IPF patient can be estimated quite accurately by an IPF-specific indirect MVV formula (FEV1 × 20.1) + 15.4. By this simple indirect MVV formula, most IPF patients would be diagnosed as having a respiratory mechanical limitation.

## Data Availability

The data-sets used and/or analyzed during the current study are available from the corresponding author on reasonable request.

## Abbreviations

CPET: Cardiopulmonary exercise test
DLCO: Diffusion capacity of the lung for Carbon Monoxide
FEV1: Forced exhaled volume in 1 s
FVC: Forced vital capacity
IPF: Idiopathic Pulmonary Fibrosis
MVV: Maximal voluntary ventilation
PH: Pulmonary Hypertension
RER: Respiration Exchange Ratio
TLC: Total Lung Capacity
VEpeak: Ventilation at peak exercise
VO2: Oxygen Uptake
